# Does Hepatitis C Virus Infection Increase Risk for Stroke? A Population-Based Cohort Study

**DOI:** 10.1371/journal.pone.0031527

**Published:** 2012-02-20

**Authors:** Chien-Chang Liao, Ta-Chen Su, Fung-Chang Sung, Wan-Hsin Chou, Ta-Liang Chen

**Affiliations:** 1 Health Policy Research Center, Taipei Medical University, Taipei, Taiwan; 2 Department of Anesthesiology, Taipei Medical University Hospital, Taipei, Taiwan; 3 Department of Cardiology, National Taiwan University Hospital, Taipei, Taiwan; 4 Department of Public Health, China Medical University, Taichung, Taiwan; Lerner Research Institute, Cleveland Clinic, United States of America

## Abstract

**Background:**

The relationship between hepatitis C virus infection and risk of stroke remains inconsistent. This study evaluates the risk of stroke in association with chronic hepatitis C infection in a longitudinal population-based cohort.

**Methods:**

We identified 4,094 adults newly diagnosed with hepatitis C infection in 2002–2004 from the Taiwan National Health Insurance Research Database. Comparison group consisted of 16,376 adults without hepatitis C infection randomly selected from the same dataset, frequency matched by age and sex. Events of stroke from 2002–2008 were ascertained from medical claims (*International Classification of Diseases, Ninth Revision, Clinical Modification,* ICD-9-CM, codes 430–438). Multivariate adjusted hazard ratios (HRs) and 95% confidence intervals (CIs) were estimated for potential associated factors including HCV infection, age, sex, low-income status, urbanization, cessation of cigarette smoking, alcohol-related illness, obesity, history of chronic diseases and medication use.

**Findings:**

During 96,752 person-years of follow-up, there were 1981 newly diagnosed stroke cases. The HRs of stroke associated with medical conditions such as hypertension, diabetes and heart disease were 1.48 (95% CI 1.33 to 1.65), 1.23 (95% CI 1.11 to 1.36) and 1.17 (95% CI 1.06 to 1.30), respectively, after adjustment for covariates. The cumulative risk of stroke for people with hepatitis C and without hepatitis C infections was 2.5% and 1.9%, respectively (p<0.0001). Compared with people without hepatitis C infection, the adjusted HR of stroke was 1.27 (95% CI 1.14 to 1.41) for people with hepatitis C infection.

**Conclusion:**

Chronic hepatitis C infection increases stroke risk and should be considered an important and independent risk factor.

## Introduction

Chronic hepatitis C virus (HCV) infection is prevalent worldwide, with approximately 170 million persons suffering from this disease [1], [2]. The epidemiology, natural history of chronic infection, virology and medical therapy for HCV infection has been well documented [2]. However, pandemic HCV infection remains a serious problem of global concern because of other long-term consequences of the infection. Patients with HCV infection may progress to cirrhosis and subsequently develop complications such as ascites, variceal bleeding, encephalopathy and hepatocellular carcinoma [1], [2].

Stroke is the second leading cause of death worldwide and the leading cause of acquired disability in adults in most regions [3]–[5]. An international multicentre population-based study has identified cardiac diseases, hypertension, diabetes, smoking, alcohol intake, unhealthy diet, abdominal obesity, lack of exercise, psychosocial stress and depression as risk factors associated with 90% of stroke risk [5]. Nevertheless, other risk factors associated with the prevalence of stroke require further study.

Substantial experimental and epidemiologic evidence has documented the potential role of HCV infection in the development and progress of carotid atherosclerosis [6]–[9]. Altered cerebral metabolism in patients with chronic HCV infection has been proposed [10], yet the association of stroke with HCV infection as a consequence of carotid atherosclerosis remains unclear. Although chronic HCV infection is considered an independent risk predictor of cerebrovascular mortality, whether HCV infection increases the incidental event of stroke is undetermined [11]. To clarify the potential impact of HCV infection on stroke, we conducted a population-based cohort study using reimbursement claims from Taiwan's National Health Insurance Research Database with a follow-up period of 4 to 7 years.

## Methods

### Study design and sample

Taiwan's National Health Insurance has documented all medical claims for insured beneficiaries since 1996. With identification numbers scrambled to protect patient privacy, information collected for this study included gender, birthday, disease codes, health care rendered, medications prescribed, admissions, discharges, medical institutions and physicians providing services. In this longitudinal cohort study with a randomly selected population of one million insured subjects, we identified patients aged 20 years and older who were newly diagnosed with HCV infection in 2002–2004 as the HCV cohort. The non-HCV cohort comprised people aged ≥20 years randomly selected from individuals without HCV infection at a ratio of 1∶4 (exposed vs. non-exposed), with frequency matching by age and sex during the same time period. Patients with previous history of stroke were excluded when establishing both cohorts. Overall, 20,470 insured adults were included for the prospective analysis. This follow-up started in 2002 to include all incident stroke cases or until censoring due to death, loss to follow-up or other causes by the end of 2008 to explore whether the HCV cohort had increased risk of developing stroke.

### Criteria and definition

*The International Code of Diseases, Ninth Edition, Clinical Modification* (ICD-9-CM) was used to identify parameters from individual health reimbursement claims. The HCV cohort consisted of patients with diagnosis of HCV infection (ICD-9-CM 070.41, 070.44, 070.51 and 070.54), and the non-HCV cohort was controls with no diagnosis of HCV infection. Both groups were treated as fixed cohorts. HCV cohort was defined as patients with primary diagnosis according to positive serum results during hospitalization or outpatient visits. New stroke cases (ICD-9-CM of 430–438) were further defined from emergency and inpatient medical records afterwards. Co-existing medical conditions such as obesity (ICD-9-CM 278), hyperlipidemia (ICD-9-CM 272.9), diabetes (ICD-9-CM 250), ischemic heart disease (ICD-9-CM 410–414) and hypertension (ICD-9-CM 401, 402, 403, 404 and 405) were considered as covariates in this study. We further considered associated alcohol-related illnesses as alcoholic psychoses (ICD-9-CM 291), alcohol dependence syndrome (ICD-9-CM 303), alcohol abuse (ICD-9-CM 305), alcoholic fatty liver (ICD-9-CM 571.0), acute alcoholic hepatitis (ICD-9-CM 571.1), alcoholic cirrhosis of liver (ICD-9-CM 571.2) and alcoholic liver damage (ICD-9-CM 571.3). In addition, subjects with history of cessation of cigarette smoking were also identified. Medication use of statins and angiotensin-converting enzyme (ACE) inhibitor during the follow-up period were also considered in this study [12], [13]. The National Health Insurance Research Database has been verified as a valid resource for population-based research [14].

### Statistical analysis

We compared the distribution of demographic factors and the proportions of comorbidities between the HCV and non-HCV cohorts. The crude incidence rates of stroke were calculated in the follow-up period until the end of 2008. The duration of observation for each beneficiary was calculated until stroke was diagnosed or the beneficiary was censored for death, migration or discontinuation of insurance coverage. Adjusted hazard ratios (HRs) with 95% confidence intervals (CIs) for HCV infection and other factors associated with stroke risk were calculated using Cox proportional hazard analyses. Two-sided probability values less than 0.05 were considered statistically significant. We considered associated factors in the univariate regressions with a p<0.2 in the multivariate Cox proportional hazard model to estimate the hazard ratios (HRs) for stroke. All analyses were performed by SAS software version 9.1 (SAS Institute Inc., Carey, NC, USA).

### Ethical Approval

Insurance reimbursement claims used in this study were from Taiwan's National Health Insurance Research Database, which is available for public access. This study was conducted in accordance with the Helsinki Declaration. To protect personal privacy, the electronic database was decoded with patient identifications scrambled for further public access for research. According to National Health Research Institute regulations, informed consent is not required due to decoded and scrambled patient identification. However, this study was evaluated and approved by Taiwan's National Health Research Institutes.

## Results

The eligible study subjects were 4094 persons in the HCV cohort and 16,376 persons in the non-HCV cohort (**Table 1**). The HCV infection cohort had a higher proportion of individuals with low incomes, living in less urbanized areas, and visiting the smoking cessation clinic compared with individuals without HCV infection. Patients with HCV infection were also more likely to have hypertension (42.4% vs. 34.9%, p<0.0001), hyperlipidemia (40.4% vs. 26.8%, p<0.0001), diabetes mellitus (25.6% vs. 13.9%, p<0.0001), ischemic heart disease (21.7% vs. 15.0%, p<0.0001), alcohol-related disease (10.3% vs. 1.5%, p<0.0001) and obesity (1.8% vs.1.1%, p = 0.0003). There were significant differences in medication use, with use of stains (p = 0.0102) and ACE inhibitor (p<0.0001) higher in the HCV group than in the non-HCV group.

**Table 1:** Comparisons in demographic characteristics and comorbidities between cohorts with and without hepatitis C infection.

	Infection with hepatitis C				
	No (N = 16,376)		Yes (N = 4094)		p-value
Sex	n	(%)	n	(%)	1.00
Female	8184	(50.0)	2046	(50.0)	
Male	8192	(50.0)	2048	(50.0)	
Age, years					1.00
20–29	1200	(7.3)	300	(7.3)	
30–39	2068	(12.6)	517	(12.6)	
40–49	3384	(20.7)	846	(20.7)	
50–59	3916	(23.9)	979	(23.9)	
60–69	3392	(20.7)	848	(20.7)	
≥70	2416	(14.8)	604	(14.8)	
Low-income	289	(1.8)	98	(2.4)	0.0082
Urbanization					<0.0001
Low	4014	(24.5)	1329	(32.5)	
Moderate	4055	(24.8)	1041	(25.4)	
High	3980	(24.3)	951	(23.2)	
Very high	4327	(26.4)	773	(18.9)	
Smoking cessation servive	315	(1.9)	133	(3.3)	<0.0001
History of disease					
Hypertension	5718	(34.9)	1736	(42.4)	<0.0001
Hyperlipidemia	4396	(26.8)	1655	(40.4)	<0.0001
Diabetes	2274	(13.9)	1047	(25.6)	<0.0001
Congestive heart disease	2451	(15.0)	888	(21.7)	<0.0001
Alcohol-related disease	252	(1.5)	421	(10.3)	<0.0001
Obesity	184	(1.1)	75	(1.8)	0.0003
Statin use	2112	(12.9)	467	(11.4)	0.0102
ACE inhibitor use	4049	(24.7)	1290	(31.5)	<0.0001

ACE, angiotensin-converting enzyme.

**Table 2** shows that the incidence of stroke was significantly higher in the HCV cohort than in the non-HCV cohort (25.3 vs. 19.3 per 1000 person-years) with a unadjusted HR of 1.30 (95% CI 1.17 to 1.44). Female gender, greater age, lower income, living in less urbanized areas, hyperlipidemia, diabetes, heart disease and hypertension were also associated with the risk of stroke before adjustment. The unadjusted HRs of risk of stroke for users of statins and ACE inhibitor were 1.32 (95% CI 1.17 to 1.49) and 2.02 (95% CI 1.85 to 2.21), respectively. Those who received smoking prevention services seemed to benefit from a decreased risk of stroke.

**Table 2:** Incidences of stroke and Cox model measured hazard ratios of stroke associated with hepatitis C infection, demographic factors and comorbidities.

				Univariate	
	Person-years	Cases	Incidence rate^a^	HR	(95% CI)
Hepatitis C					
No	77686	1499	19.3	1.00	(reference)
Yes	19066	482	25.3	1.30	(1.17–1.44)
Sex					
Female	48830	1069	21.9	1.00	(reference)
Male	47922	912	19.0	0.87	(0.80–0.95)
Age, years					
20–29	7413	11	1.5	1.00	(reference)
30–39	12806	44	3.4	2.31	(1.19–4.48)
40–49	20804	160	7.7	5.17	(2.81–9.53)
50–59	23637	414	17.5	11.8	(6.48–21.5)
60–69	19690	645	32.8	22.1	(12.2–40.1)
≥70	12403	707	57.0	38.5	(21.2–69.9)
Low-income	1689	60	35.5	1.75	(1.36–2.27)
Urbanization					
Low	24636	686	27.8	1.58	(1.40–1.79)
Moderate	24258	468	19.3	1.10	(0.96–1.25)
High	23530	400	17.0	0.97	(0.85–1.11)
Very high	24328	427	17.6	1.00	(reference)
Smoking cessation servive	2256	23	10.2	0.49	(0.33–0.74)
Alcohol-related illness	3042	69	22.7	1.10	(0.87–1.40)
Obesity	1276	24	18.8	0.92	(0.61–1.37)
History of hyperlipidemia	29059	749	25.8	1.41	(1.29–1.55)
History of diabetes	15162	576	38.0	2.20	(2.00–2.42)
History of heart disease	15029	641	42.7	2.60	(2.36–2.85)
History of hypertension	34095	1270	37.2	3.28	(2.99–3.59)
Statin use	12644	328	25.9	1.32	(1.17–1.49)
ACE inhibitor use	25010	820	32.8	2.02	(1.85–2.21)

a Per 1000 person-years.

ACE, angiotensin-converting enzyme; CI, confidence interval; HR, hazard ratio.

After adjustment, HCV infection demonstrated a HR of 1.38 (95% CI 1.24 to 1.53) for stroke, or of 1.27 (95% CI 1.14 to 1.41) in the multivariate model (**Table 3**). Age (HR 1.68, 95% CI 1.62 to 1.74), low income (HR 1.47, 95% CI 1.14 to 1.90), history of diabetes (HR 1.23, 95% CI 1.11 to 1.36), ischemic heart disease (HR 1.17, 95% CI 1.06 to 1.30) and hypertension (HR 1.48, 95% CI 1.06 to 1.30) were also significant and independent factors associated with increased risk of stroke. After adjustment, the use of statins (HR 0.79, 95% CI 0.69 to 0.90) and ACE inhibitor (HR 0.75, 95% CI 0.67 to 0.83) were associated with reduced risk of stroke.

**Table 3:** Multivariable Cox model measured hazard ratios and 95% confidence intervals for stroke.

		Multivariate-adjusted	
		HR	(95% CI)
Hepatitis C	yes vs. no	1.22	(1.13–1.40)
Male	vs. female	1.00	(0.91–1.09)
Age	10-year increment	1.05	(1.05–1.06)
Low-income	yes vs. no	1.52	(1.17–1.96)
Urbanization	vs. very high	(Not included, p>0.2)	
Low			
Moderate			
High			
Smoking cessation services	yes vs. no	0.64	(0.42–0.97)
Alcohol-related illness	yes vs. no	(Not included, p>0.2)	
Obesity	yes vs. no	(Not included, p>0.2)	
History of hyperlipidemia	yes vs. no	0.96	(0.87–1.07)
History of diabetes	yes vs. no	1.37	(1.23–1.52)
History of heart disease	yes vs. no	1.25	(1.13–1.39)
History of hypertension	yes vs. no	1.77	(1.57–1.99)
Statin use	yes vs. no	0.79	(0.69–0.90)
ACE inhibitor use	yes vs. no	0.75	(0.67–0.83)

ACE, angiotensin-converting enzyme; CI, confidence interval; HR, hazard ratio.

## Discussion

In this population-based cohort study, we found that chronic HCV infection was associated with significantly increased risk of stroke after controlling for conventional stroke risk factors. This further upgrades previous associated findings that chronic HCV infection might be linked with carotid atherosclerosis [6]–[9], [15], [16] and acute myocardial infarction [17]. To the best of our knowledge, our study is the first to investigate the relationship between HCV infection and stroke event in a population-based longitudinal cohort.

Previous studies have reported positive relationships between HCV infection and carotid intima-media thickness, plaque and stroke. The association varied in populations with different prevalence of HCV infection, risk factors and study design [6]–[9], [18]–[21]. A cross-sectional study supported the possible link between HCV infection and carotid atherosclerosis in subjects without severe liver dysfunction [20]. A prospective population-based study also suggested that chronic infection plays an important role in human carotid atherogenesis [22]. Positive correlations between HCV infection, carotid atherosclerosis and cardiovascular diseases also have been investigated in specific patient populations. Among patients with type 2 diabetes, significant association between HCV infection and ultrasonographic-evident carotid atherosclerosis was noted [7]. HCV infection is also closely associated with increased aortic stiffness and cardiovascular events in dialysis patients [22]. In addition, HIV-infected individuals with HCV co-infection were found to have increased risk of cardiovascular disease and acute myocardial infarction [18], [19]. Aslam's research review concluded that HCV-positive subjects had higher incidence of carotid atherosclerotic plaques compared to HCV-negative individuals [8].

Chronic HCV infection *per se* could be considered a chronic inflammatory process that might play a role in the pathogenesis of carotid arterial remodelling [7]. Localization of RNA of HCV in human carotid plaques provides strong evidence for an association between HCV infection and atherosclerosis [6]. Forton et al. hypothesized that HCV infection might be related to cerebral dysfunction, particularly neuropsychological symptoms and cognitive impairment [23]. Furthermore, chronic HCV infection has potential effects on cerebral metabolism which could not be explained by hepatic encephalopathy or a drug-induced insult [10]. However, further large-scale prospective studies are needed to investigate these findings and hypotheses.

Controversy persists about correlation between HCV infection and carotid atherosclerosis or cardiovascular disease [15]–[17]. A cross-sectional study conducted by Völzke et al. suggested that serum-positivity to hepatitis B surface antigen or hepatitis C antibody was not associated with atherosclerotic signs such as myocardial infarction, stroke and carotid plaques [16]. However, several important limitations were involved in that study, including inadequate and temporal serologic markers of HCV infection and a cross-sectional, not longitudinal, study design. No association between HCV infection and acute myocardial infarction was found in a case-control study in the US Army [17]. Similarly, Bilora et al. proposed no evidence associating HCV infection and carotid atherosclerosis [15]. Another longitudinal study also failed to confirm the association between chronic active hepatitis B/C and carotid plaques due to its poorly defined diagnosis of serostatus [22]. Different study populations, limited sample size with traditional risk factors of atherosclerosis and cerebrovascular disease, various controlled risk factors and prevalence of HCV infection all might contribute to these previous studies' inconsistent findings.

A recent community-based prospective cohort study concluded that chronic HCV infection is an independent risk predictor of cerebrovascular deaths, showing a severity-dependent cerebrovascular mortality with increasing serum HCV RNA level [11]. However, that study was limited by non-validated data derived from the diagnosis on death certificate addressed by administrative instead of medical personnel [11]. The previous study also did not consider the effects of cardiovascular medication use on risk of stroke [12], [13]. As a population cohort, our findings suggest a positive association between chronic HCV infection and stroke event which was identified from National Health Insurance records after the adjustment for sociodemographic factors, history of diseases and cardiovascular medication use. Our study is the first to report the association of HCV infection and increased risk of stroke [14].

Our study differs from previous research on HCV infection and stroke in several aspects. Retrospective exclusion of individuals with previous medical history of stroke helps us define fresh cases and clarify the correlation between HCV infection and development of stroke. In addition, considering the major cardiovascular risk factors for the data analysis, we could examine the effects of HCV infection independently on stroke with risk confounders. Several studies have positively linked HCV infection with type 2 diabetes and metabolic syndrome, which are important risk factors for stroke [23]–[26]. Even though type 2 diabetes and hypertension may increase the risk of stroke, this study demonstrated an independent risk of stroke after controlling for diabetes and hypertension in multivariate analyses. These results further suggest HCV infection plays an independent and unique role in incident stroke.

Our study has several limitations. Sub-clinical HCV-infected patients might be included among our non-HCV cohort. The history of hypertension, diabetes, hyperlipidemia, ischemic heart disease, alcohol-related diseases and obesity was based on medical claims in this study and might be underestimated. Moreover, our study has no data for patient lifestyle associated with stroke. This study mainly focused on clinical end points as detailed by medical reimbursement claims, rather than on underlying pathologic lesions in vessels. In addition, this study could not provide a disease severity-dependent relationship between HCV infection and stroke. We postulate that there might be a dose-response (titer-dependent) relationship between serum HCV RNA levels and risk of stroke event [11].

In conclusion, we identified an independent association between chronic HCV infection and risk of stroke after controlling for traditional stroke risk factors with a population-based longitudinal cohort. Our data suggest a need for regular screening for cardiovascular risk factors and preclinical atherosclerosis among patients with HCV infection for early prevention of atherosclerotic disease and stroke.
